# Supplementary material for: Ether anesthetics prevents touch-induced trigger hair calcium-electrical signals excite the Venus flytrap
Source: Sci Rep. 2022 Feb 18;12:2851. doi: 10.1038/s41598-022-06915-z (PMC8857258; doi:10.1038/s41598-022-06915-z)
Supplement: Supplementary file 1 — Supplementary Information. [file 41598_2022_6915_MOESM1_ESM.pdf]

Supplementary information

Supplemental Figures

Fig. S1

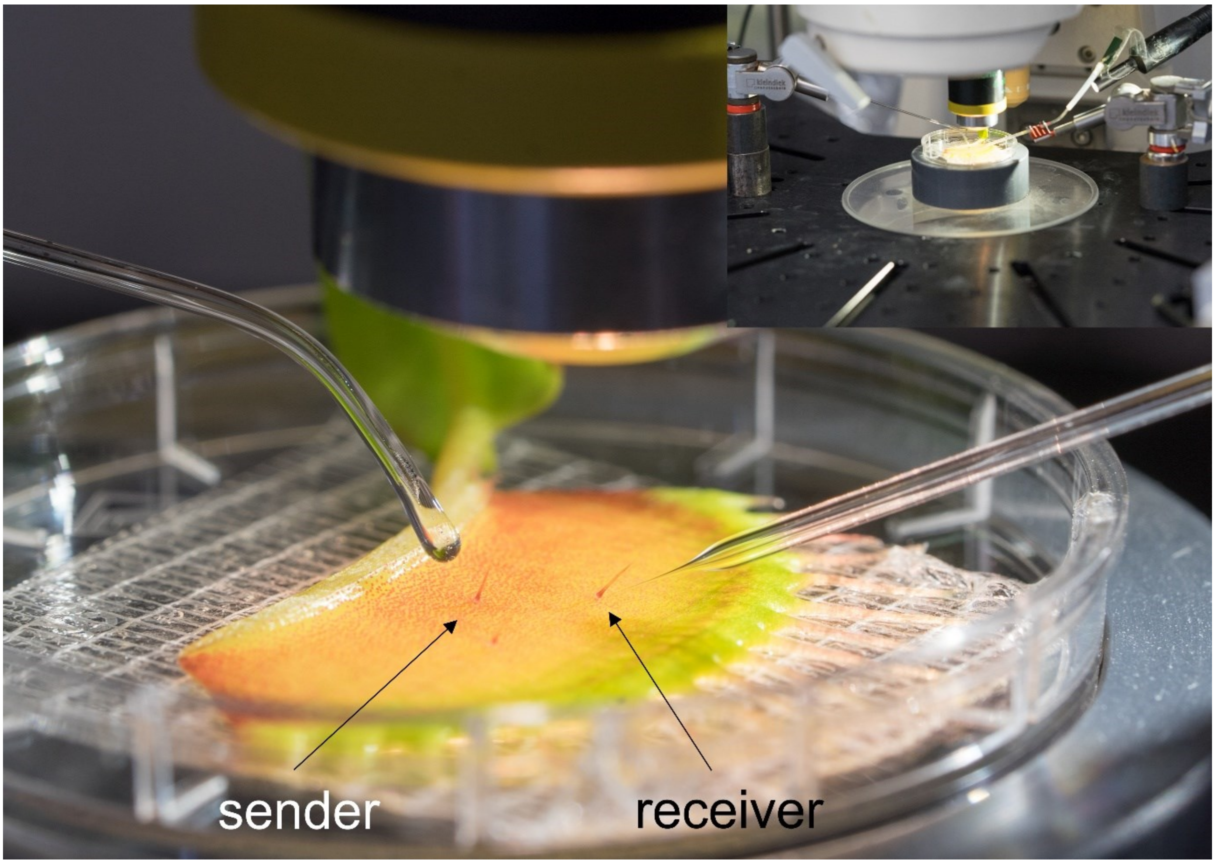

Exemplary setup of the voltage recording. The trap half is fixed under the microscope (upper right). While one trigger hair (sender) can be stimulated from the left, another hair (receiver) is impaled from the right.

Fig. S2

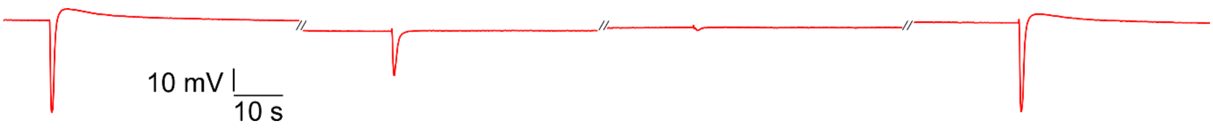

Representative surface potential measurement of a *Dionaea* trap under an ether atmosphere as seen in Supplementary Movie S4, with following ventilation.

Fig. S3

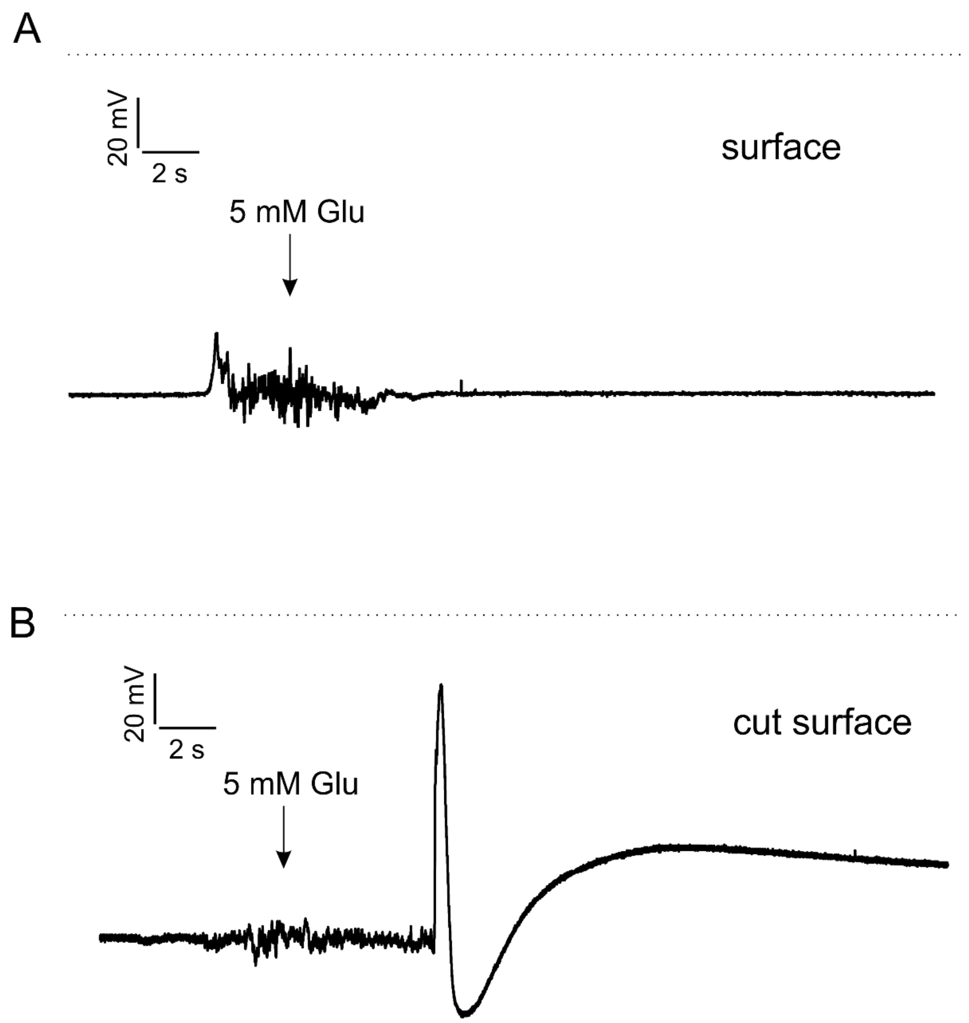

Membrane potential of a *Dionaea* trap where 5 mM glutamate (arrow) was applied to the undamaged surface (up) ore the cut surface (down). Only application to the cut surface initiated an AP. Dotted line represents 0 mV.

Fig. S4

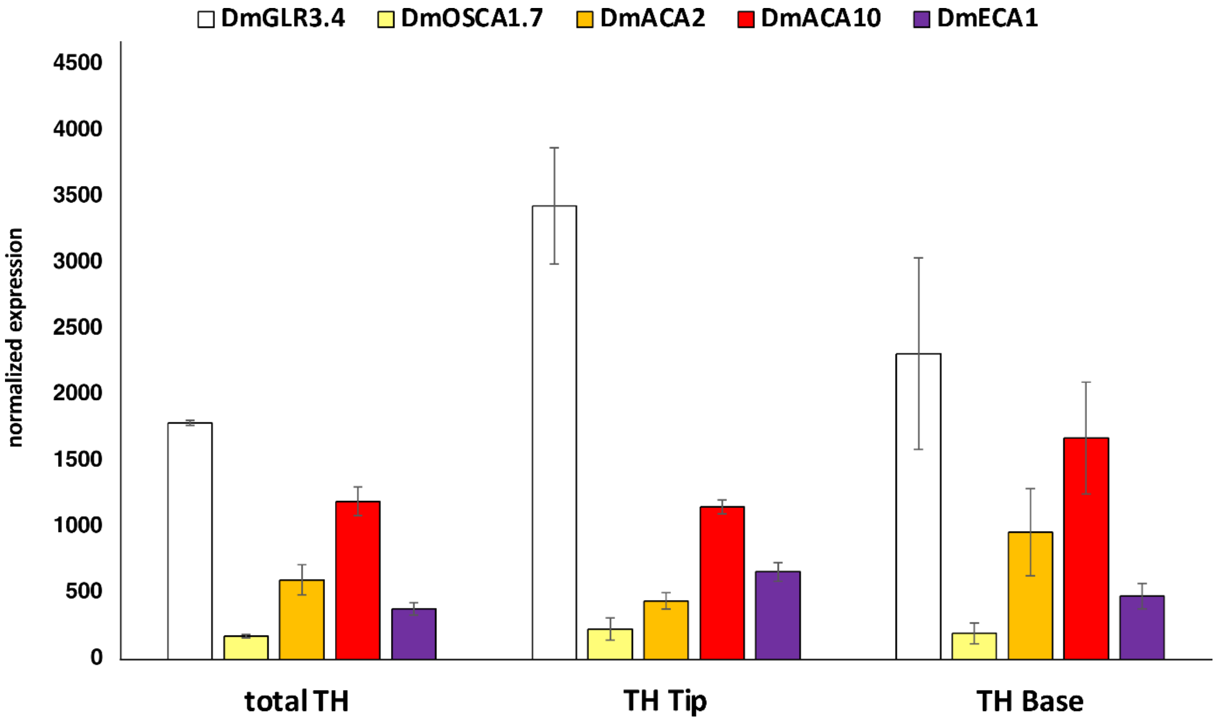

Normalized expression values of selected transporters quantified by qPCR in the whole trigger hair, in the tip/lever of the trigger hair, and in the base/podium of the trigger hair (mean normalized to 10,000 actin ± SE; n = 3).

Supplemental Table S1

Gene (ID)	Identifier	TH specific expression	Podium (enriched) expression	Enriched in trap stage V vs VI	Enriched in trigger hair stage V vs VI
DmMSL10	Dm_00009130-RA	+	+	-	(+)
KDM1	Dm_00004067-RA	+	+	+	+
DmSKOR	Dm_00007946-RA	+	+	+	+
GLR3.4	Dm_00004609-RA	+	-	+	+
GLR3.6	Dm_00002270-RA	+	+	+	+
ECA1	Dm_00014557-RA	-	-	-	-
ACA2	Dm_00018589-RA	+	+	-	-
ACA10	Dm_00009018-RA	-	+	(+)	(+)
DmOSCA1.7	Dm_00005287-RA	-	-	+	+

Supplemental Movies

Similar results were obtained from at least three biologically independent GCaMP6f expressing plants for all shown Supplementary movies.

Movie S1:

Representative movie of a GCaMP6f expressing *Dionaea* trap where the trigger hair is mechanical stimulated. The first stimulus (6 s) was subthreshold, while the following (7 s) was initiating a Ca^{2+} signaling.

Movie S2:

Young *Dionaea* traps without the characteristic pigmentation also show a Ca^{2+} signal in the digestive glands after mechanical stimulation.

Movie S3:

10x time-lapse video of a *Dionaea* trap stimulated once per minute. After minute 3, a saturated ether atmosphere was created for 5 minutes, which was then replaced with fresh air. Please note that even under full anesthesia only the stimulated trigger hair podium shows an unaffected Ca^{2+} signal.

Movie S4:

Setup used for surface potential measurements as presented in Supplementary Fig. S2.

Movie S5:

Juvenile stage 5 trap mechanically stimulated with a soft brush. Please note that the Ca^{2+} signal does not spread over the whole trap but remains localized.

Movie S6:

A *Dionaea* trap mechanically stimulated with a fine brush on the trigger hair on the left and pressure applied with a pipette tip on the right. Please note that both stimulations elicit a fast Ca^{2+} signaling in the entire trap, but the calcium signal at the site of the pressure decays very slowly.

Movie S7:

Higher resolution of intense wounding leading to a particularly strong calcium signal in the vasculature.

Movie S8:

10x time-lapse video of a trap where 5 mM glutamate was applied on the trap surface (3 s) not leading to any calcium signaling. Only application on the cut midrib (9 s) resulted in rapid and long-lasting calcium signaling, especially in the vasculature. As a control, a trigger hair was mechanically stimulated at 18 s, which resulted in a regular calcium signal.

Movie S9:

10x time lapse of a trap that was mechanically stimulated once per minute. After 3 min, ether was added. When calcium signal transduction failed in the trap, strong injury was applied at 30 sec. Please note that again under anesthesia mechanical stimulation still resulted in a calcium signal in the podium and also the injury did not result in a calcium signal propagation in the trap.

Movie S10:

10x time lapse of two *Dionaea* traps side by side. In both traps, a mechanical control AP was triggered at 6 s. Afterwards, only the right trap was treated with ether and after 8 minutes (54 s) a hair was stimulated again to verify that the right trap was anaesthetized. Subsequently (100 s), 5 mM glutamate was applied to the incised midrib in both traps, resulting in a Ca^{2+} signal only in the untreated left trap. Even a new application of 20 mM glutamate (72 s) to the anaesthetized trap did not lead to a calcium signal.
